# New Therapy of Skin Repair Combining Adipose-Derived Mesenchymal Stem Cells with Sodium Carboxymethylcellulose Scaffold in a Pre-Clinical Rat Model

**DOI:** 10.1371/journal.pone.0096241

**Published:** 2014-05-02

**Authors:** Cristiano Rodrigues, Adriano M. de Assis, Dinara J. Moura, Graziele Halmenschlager, Jenifer Saffi, Léder Leal Xavier, Marilda da Cruz Fernandes, Márcia Rosângela Wink

**Affiliations:** 1 Laboratórios de Pesquisa em Biologia Celular, Departamento de Ciências Básicas da Saúde, Universidade Federal de Ciências da Saúde de Porto Alegre, Porto Alegre/RS, Brazil; 2 Genética Toxicológica, Departamento de Ciências Básicas da Saúde, Universidade Federal de Ciências da Saúde de Porto Alegre, Porto Alegre/RS, Brazil; 3 Estresse Oxidativo e Poluição Atmosférica, Departamento de Ciências Básicas da Saúde, Universidade Federal de Ciências da Saúde de Porto Alegre, Porto Alegre/RS, Brazil; 4 Programa de Pós-Graduação em Ciências Biológicas: Bioquímica, Departamento de Bioquímica, Universidade Federal do Rio Grande do Sul, Porto Alegre/RS, Brazil; 5 Laboratório de Biologia Celular e Tecidual, Departamento de Ciências Morfofisiológicas, Pontifícia Universidade Católica do Rio Grande do Sul, Porto Alegre/RS, Brazil; 6 Laboratório de Pesquisa em Patologia, Departamento de Ciências Básicas da Saúde, Universidade Federal de Ciências da Saúde de Porto Alegre, Porto Alegre/RS, Brazil; University of KwaZulu-Natal, South Africa

## Abstract

Lesions with great loss of skin and extensive burns are usually treated with heterologous skin grafts, which may lead rejection. Cell therapy with mesenchymal stem cells is arising as a new proposal to accelerate the healing process. We tested a new therapy consisting of sodium carboxymethylcellulose (CMC) as a biomaterial, in combination with adipose-derived stem cells (ADSCs), to treat skin lesions in an *in vivo* rat model. This biomaterial did not affect membrane viability and induced a small and transient genotoxicity, only at the highest concentration tested (40 mg/mL). In a rat wound model, CMC at 10 mg/mL associated with ADSCs increased the rate of cell proliferation of the granulation tissue and epithelium thickness when compared to untreated lesions (Sham), but did not increase collagen fibers nor alter the overall speed of wound closure. Taken together, the results show that the CMC is capable to allow the growth of ADSCs and is safe for this biological application up to the concentration of 20 mg/mL. These findings suggest that CMC is a promising biomaterial to be used in cell therapy.

## Introduction

The skin is the largest organ of the human body and its integrity allows body functions such as elimination of toxins, resistance to mechanical stress, prevention of water loss, participation in immune responses and protection against external agents [1], [2], [3]. However, many factors and systemic conditions may compromise the dermal function, such as, diabetes mellitus, trauma, inflammatory diseases, metabolic abnormalities, bleeding disorders, smoking, immunosuppression, malnutrition and obesity [1]. Therefore, promotion of skin lesion healing, regardless of the nature of the causative agent, is of utmost importance [4].

After injury to skin, a new epithelium has to form in order to close the wound and restore the barrier function. This requires the proliferation and directional migration of keratinocytes, fibroblasts and mesenchymal stem cells (MSCs) to wound edges [5], [6]. MSCs are essential for skin homeostasis and repair of the lesions, by promoting cell differentiation, immune-modulation, secretion of growth factors to drive re-epithelialization and neovascularization and modulating resident stem cells [7], [8]. Both, proliferation and migration of these cells are controlled by extracellular hormones, e.g., glucocorticoids and paracrine factors [5], [6], which provides attractive opportunities for therapeutic interventions.

In the last decade, studies have indicated that human adipose tissue contains populations of MSCs, called adipose-derived stem cells (ADSCs) [9]. In contrast to stem cells obtained from bone marrow (BM-MSCs), whose access can be painful for the patient, requiring general or spinal anesthesia and most often resulting in low numbers of MSCs after processing [10], ADSCs can be collected from adipose tissue, as the waste material after liposuction in aesthetic surgeries [11]. It is estimated that 1 mL of lipoaspirate contains at least 7.3×10^3^ fibroblast colony forming (CFU-F), corresponding to 0.5–5% of the total population of stromal vascular fraction (SVF) (4.04±3.26 on average). Therefore, the SVF adipose tissue contains a quantity of CFU-F, 400 times higher than the bone marrow, where the frequency of CFU-F is estimated to be about 1 per 10^5^ cells [12].

Thus, the possibility of deriving ADSCs from adult adipose tissues, the relatively easy protocols for their isolation and expansion in culture, and the fact that they are immunologically well tolerated, allowing the transplant from one individual to another of the same species, makes them attractive candidates for new studies in translational clinical research [9].

Besides the availability and abundance of ADSCs in the body, several steps remain to be solved before these cells become effectively and safely used as an established therapy. One of the obstacles is to find the correct substances or molecules capable to offer the correct environment to these cells, allowing their safe deployment in tissues or lesions. The long-term success of these therapies requires biocompatibility of the biomaterial that hosts the tissue while retaining the functionality of the cells within the implanted tissue [13]. Sodium carboxymethylcellulose (CMC) is synthesized by the hydration of the cellulose with sodium hydroxide and from an alkaline pulp catalyzed reaction with chloroacetic acid [13]. This synthetic polymer has been shown to be suitable for the protection and treatment of skin wounds, including partial thickness burns [14]. It has a strong ability to absorb and transport fluids and protect from bacterial exposure of the external environment [14]. These attributes make CMC suitable for use as a polymer matrix for wound healing, although its toxicity was not yet fully evaluated [15]. Over the years, CMC has been used as a single material with or without combination with drugs and co-excipients in partial thickness wounds, diabetic ulcers, lesions of the foot and deep dermal filler [15].

Considering that ADSCs have a great potential for clinical use in tissue repair and regeneration, but its efficacy depends of biomaterials that allow the proper tissue-engineering products, the aim of this study was to test the applicability of CMC as a scaffold for the ADSCs, to treat dermal lesions in a pre-clinical model.

## Materials and Methods

### Ethical Aspects

The protocols used in this study were approved by the Ethics Committee on Animal Use (CEUA) of Universidade Federal de Ciências da Saúde de Porto Alegre (UFCSPA), under the number **10-009**, following the resolutions of the the CONCEA (Conselho Nacional de Controle de Experimentação Animal). The NIH “Guide for the Care and Use of Laboratory Animals” (NIH publication N° 80–23, revised 1996) was followed in all experiments. All surgery was performed under ketamine and xylazine anesthesia and all efforts were made to minimize the animals suffering.

### Chemicals

Dulbecco’s modified Eagle medium low glucose (DMEM), Ca^2+^ and Mg^2+^ free Hank’s balanced salt solution containing 10 mM sodium HEPES (HB-CMF-HBSS), collagenase Type I, trypsin–EDTA, penicillin/streptomycin and carboxymethylcellulose sodium (medium viscosity) were obtained from Sigma Chemical Co. (St. Louis, M.O., USA); fetal bovine serum (FBS) from Cultilab (São Paulo, S.P., Brazil); trypan blue (TB) was purchase from GIBCO (Grand Island, N.Y., USA). The measurement of the lactate dehydrogenase activity was determined by commercial kit (Labtest, Lagoa Santa, M.G., BR). Low-melting point agarose and agarose were obtained from Invitrogen (Carlsbad, C.A., USA).

### Extraction and Cultivation of ADSCs

The ADSCs were extracted from visceral adipose tissue of Wistar rats (6–8 weeks), as previously described [16]. Briefly, the abdominal fat tissue from 2 rats was transferred to a petri dish, washed with Hanks solution and dissociated mechanically with a scalpel. The fat fragments were transferred to a Falcon tube containing 3 mg/mL collagenase Type I solubilized in DMEM medium without FBS and incubated in a water bath (37°C) for 45 minutes (being vortexed every 15 minutes). After this time twice the volume of DMEM supplemented with 10% FBS was added, to inactivate the collagenase. Cells were centrifuged for 10 minutes at 1000 rpm. The supernatant was discarded and cells were resuspended in a known volume of DMEM. Cell count was performed microscopically, and cell viability was assessed by trypan blue exclusion. 5×10^6^ cells/cm^2^ were seeded in six-well plates and cultured at 37°C in an incubator with 5% CO_2_ in the presence of 3 mL DMEM low glucose medium supplemented with FCS 10%, penicillin (100 U/mL), streptomycin (100 mg/mL). After 24 hours of incubation, half of the culture medium was removed and replaced with fresh medium. At the end of 72 hours, the culture medium was removed completely and the non-adherent fraction was subtracted. The attached fraction was maintained until confluence, generally the fourth passage, when cells were harvested to be used in experiments.

### Osteogenic and Adipogenic Differentiation of ADSCs

Cell differentiation was performed according the technique already described [17]. Osteogenic differentiation was induced by culturing ADSCs for up to 8 weeks in DMEM supplemented with 10^–8^ M dexamethasone, 5 µg/mL ascorbic acid 2-phosphate and 10 mM β-glycerophosphate. In order to observe calcium deposition, cultures were washed once with PBS, fixed with 4% paraformaldehyde in PBS for 15–30 minutes, and stained for 5 minutes with Alizarin Red S stain at pH 4.2. Excess of stain was removed by several washes with distilled water. To induce adipogenic differentiation, ADSCs were cultured for up to 8 weeks in DMEM supplemented with 10^–8^ M dexamethasone, 2.5 µg/mL insulin, 100 µM indomethacin and, in some experiments, 3.5 µM rosiglitazone. Adipocytes were easily discerned from the undifferentiated cells by phase-contrast microscopy. To further confirm their identity, cells were fixed with 4% paraformaldehyde in PBS for 1 hour at RT and stained with Oil Red O solution (three volumes of 3.75% Oil Red O in isopropanol plus two volumes of distilled water).

### Immunophenotyping Analysis

The ADSCs were characterized to confirm the presence or absence of MSC surface markers using flow cytometry. The cells were trypsinized, washed twice with PBS, centrifuged and incubated for 25 minutes at 4°C with the antibodies described in the Table 1. Excess of antibody was removed by washing with PBS. The cells were analyzed using a FACScalibur cytometer equipped with 488 nm argon laser (Becton Dickinson, San Diego, CA) and with the CellQuest software. Gating was set by using unstained cells and at least 10,000 events were collected [18].

**Table 1:** List of Antibodies.

Primary antibody	Host	Dilution	Supplier	Cat. N°
CD45 - PE	Mouse	1∶1000	Invitrogen	MR6404
CD29 - PE	Mouse	1∶1000	Invitrogen	A14886
CD90.1 - PerCP Cy5.5	Mouse	1∶1000	Invitrogen	A14798
CD11b*	Mouse	1∶1000	Invitrogen, USA	MR6200

*The unconjugated antibody CD11b was incubated with the secondary Anti-Mouse IgG − FITC antibody from Sigma Chemical Co. (St. Louis, M.O., USA) for 25 minutes

### Scaffold Preparation

Sterile sodium carboxymethylcellulose (CMC) salt of medium viscosity was diluted in laminar flow with the aid of magnetic stirrer and glass rod in low glucose DMEM medium, previously supplemented with 3.7 g of sodium bicarbonate and 2.5 g of HEPES per liter and 10% FBS. The CMC was diluted to achieve the concentrations of 10 mg/mL, 20 mg/mL and 40 mg/mL and was used to test different viscosities and behavior of cells in culture.

### Lactate Dehydrogenase Activity Assay

MSCs on the 5^th^ passage were plated with 3 mL of DMEM without phenol red in each well of 6-well plates (in triplicate), and allowed to growth until reaching a 70% confluence. Then, cells were treated with CMC at the concentrations of 10, 20 and 40 mg/mL. As a negative control we used cells cultured with standard culture medium, and as a positive control, cells lysed with Triton X-100 1%. After 24 hours or five days of growth in contact with CMC, the LDH cytotoxicity assay was performed following the manufacture protocol. The LDH activity was quantified at absorbance of 500 nm by a multiwell spectrophotometer (SpectraMax Plus Microplate Spectrophotometer, Molecular Devices, US). LDH activity in each cell supernatant was expressed by comparing it to the total enzyme activity in cells lysed with 1% of Triton X-100.

### The Alkaline Comet Assay

The alkaline comet assay was performed as described in literature with minor changes [19], [20]. After treatment of the cells with the biomaterial, cells were washed with ice-cold PBS, trypsinized with 100 µL trypsin (0.15%) and re-suspended in complete medium. Next, 20 µL of cell suspension (∼10^6^ cells/mL) was suspended in 0.75% low-melting point agarose and immediately spread onto a glass microscope slide pre-coated with a layer of 1% normal melting point agarose. The slides were incubated in ice-cold lysis solution (2.5 M NaCl, 10 mM Tris, 100 mM EDTA, 1% Triton X-100 and 10% DMSO, pH 10.0) at 4°C for a minimum of 1 hour to remove cellular proteins. The slides were then placed in a horizontal electrophoresis box containing a freshly prepared alkaline buffer (300 mM of NaOH and 1 mM of EDTA, pH ∼13.0) at 4°C for 20 minutes to allow DNA unwinding. DNA electrophoresis was performed with a 300 mA and 25 V (0.90 V/cm) electric current for 20 minutes. The slides were then neutralized (0.4 M of Tris, pH 7.5), stained with silver nitrate as described in literature and analyzed using an optical microscope [21]. Images of 100 randomly selected cells (50 cells from each of two replicate slides) were analyzed for each test of different substance concentration. Cells were scored visually into five classes, according to tail size (from undamaged - 0, to maximally damaged - 4), and a damage index (DI) value was assigned to each comet according to its class. Visual scoring of comets is a valid evaluation method determined by international guidelines and recommendations for the comet assay [22]. The DI is an arbitrary score calculated for cells in different damage classes that were visually scored by measuring the DNA migration length and the amount of DNA in the tail. The DI ranged from 0 (no tail: 100 cells × 0) to 400 (with maximum migration: 100 cells × 4).

### Micronucleus Assay

The micronucleus assay was performed according to Bonacker et al. (2004) with minor modifications. After treatment, cultures were washed twice with the media, and cytochalasin B (Cyt-B) was added at a final concentration of 2 µg/mL. Cultures were harvested 36 hours after Cyt-B addition. Cells were trypsinized and the cellular suspension was centrifuged at 2000 rpm for 5 minutes. Cells were then resuspended in 75 mM KCl solution and maintained at 4°C for 3 minutes (mild hypotonic treatment). Subsequently, the cells were centrifuged and a methanol/acetic acid (3∶1) solution was gently added. This fixation step was repeated twice and cells were then resuspended in a small volume of methanol/acetic acid and dropped onto clean slides. The fixed cells were hydrolyzed in HCl and stained according to the Feulgen method [23]. Slides were mounted and coded prior to analysis. Micronuclei were counted in 1000 binucleated cells (BNCs) with well-preserved cytoplasms. The identification of micronuclei was carried out according to described in literature [24].

### Skin Wound Model and Tissue Preparation

We used a total of 16 adult male Wistar rats (8 weeks), to avoid the influence of hormonal changes that could cause interferences in the healing process. The dorsal region was chosen to avoid animal access to their own wound and animals were housed one per box to prevent cross-access to the lesions. The animals were anesthetized with an intraperitoneal injection of ketamine and xylazine (90 mg/kg and 10 mg/kg, respectively), followed by shaving the dorsal region [25]. Then, with a punch, the animals were subjected to four circular lesions, measuring 7 mm of diameter, two upper and two lower, comprising dermis and epidermis. Immediately afterwards, the animals received topical treatment in the following order: Injury upper left (lesion 1): 100 µL of CMC 20 mg/mL+26×10^6^ of ADSCs; injury upper right (lesion 2): 100 µL of CMC 10 mg/mL+26×10^6^ of ADSCs; injury bottom left (lesion 3): 100 µL of CMC 20 mg/mL and injury bottom right (lesion 4): Sham, untreated. After the procedure, to ensure that the biomaterial stays housed in the lesion, the wounds were bandaged with polyurethane from 3 M (St. Paul, M.N., USA), permeable to oxygen, but acting as a barrier to liquids and bacteria. This bandage normally fell off naturally in the first or second day after surgery. After topical treatment, to analyze the wound healing, the animals were randomly organized in 4 groups, with 4 rats each: Group 1, the animals were euthanized four days after surgery; group 2, eight days after surgery; group 3, twelve days after surgery and group 4 were euthanized sixteen days after surgery. Animals were euthanized in CO_2_ chamber and the lesions were removed with a scalpel, keeping 2 mm of intact skin around. Tissues were stored in phosphate buffered formaldehyde 4% for subsequent histological analysis.

### Determination of Lesion Area

Lesions were photographed every day, next to one scale, using Sony Cyber Shot camera (Full HD 1080, 7.2 mega pixels, DSC-W120) and the area of lesion was measured using Image Pro Plus 6.1 software (IPP 6.1) (Media Cybernetics, CA, USA), after calibration.

### Immunostaining of Cytokeratin

The tissue was included in paraffin being sectioned into slices of 4 µM and de-paraffinized. After, the slides were placed in sodium citrate pH 6.0, heated at 92°C for 40 minutes, with drawn to cool to room temperature, for 20 minutes and then passed into distilled water for antigen retrieval. The endogenous peroxidase was blocked by placing the slides in a mixture of 95 mL of methanol plus 5 mL of hydrogen peroxide 30 V for 5 minutes, protected from light. The tissues were washed twice with distilled water and then with PBS for 5 minutes, under stirring. To block the unspecific bindings, the samples were incubating with a 1% BSA, for 30 minutes. Subsequently, each slide was incubated with a volume of 50 mL of PBS plus a primary antibody at a dilution of 1∶1000. The incubation occurred for 30 minutes at room temperature, and after at 4°C overnight. The next day, the slides were washed in PBS twice for 5 minutes and incubated with the secondary antibody (DAKO kit ADVANCE HPR), applied on each slide for 40 minutes at room temperature. Samples were washed with PBS twice for 5 minutes and incubated with tertiary antibody of the same kit. Revelation was performed with chromogen substrate (DAB), dehydration twice for 5 minutes, with xylene and assembled with Entellan. Reaction product of immunohistochemistry for cytokeratin was measured semi-quantitatively, by densitometry, using a Olympus BX-50 microscope, optical lens (10X/0.30 Ph1-UplanFI), coupled to a Motican 2500 camera and Image Pro Plus Software 6.1 (IPP 6.1) (Media Cybernetics, C.A., USA). The background correction and background staining subtraction were performed in accordance with a previously described protocol [26]. To measure optical density, at least two sections were analyzed in each case. Four images were obtained per section and four areas of interest (AOIs) were laid over the cytoplasmatic portions of epithelium in each digitized image. The epithelium thickness was measured in the same images, using an IPP 6.1 tool. At least four measurements were done in each image.

### Epithelium Thickness

Slides with hematoxylin-eosin staining were also prepared in order to measure the epithelium thickness. This measurement was performed using Image Pro Plus 6.1 software (IPP 6.1) (Media Cybernetics, C.A., USA), after adjustment of calibration blade. For acquisition was used BX-50 Olympus microscope with optical lens (10X/0.30 Ph1-UplanFI), coupled to a camera Motican 2500. For each wound of each animal, two images were made, and four measurements for each image.

### Collagen Fibers Analysis

After the preparation of histological slides, we used the staining of picrosirius, in order to examine whether the treatment used for healing was able to change the number of collagen fibers [27]. The images were taken with optical lens Olympus BX-50 microscope (10X/0.30 Ph1-UplanFI), using polarizing filter to highlight the collagen Type I and Type III on a black background. For each lesion of each animal, three fields of six sections were captured, using microscope images software DP2-BSW ver. 2.2 (Build 6212). The analysis was performed by the Image Pro Plus 6.1 software, proceeding with the marking of pixels according to their birefringence pattern (greenish/yellow-greenish or orange, orange-reddish). Quantification was performed on the percentage histogram of the software itself, totaling 16 points through injury.

### Statistics

The toxicity experiments were repeated three times independently. The results were expressed as mean ± SD. Data were analyzed by one-way variance (ANOVA) for the toxicological analysis and two-way for histological parameters. The means were compared by Tukey’s test for multiple comparisons, with p<0.05 considered statistically significant. Statistical analysis was performed using Prism 4.0 software (GraphPad, La Jolla, CA).

## Results

### Confirmation of the ADSC Stem Cell Character

**Figure 1:**
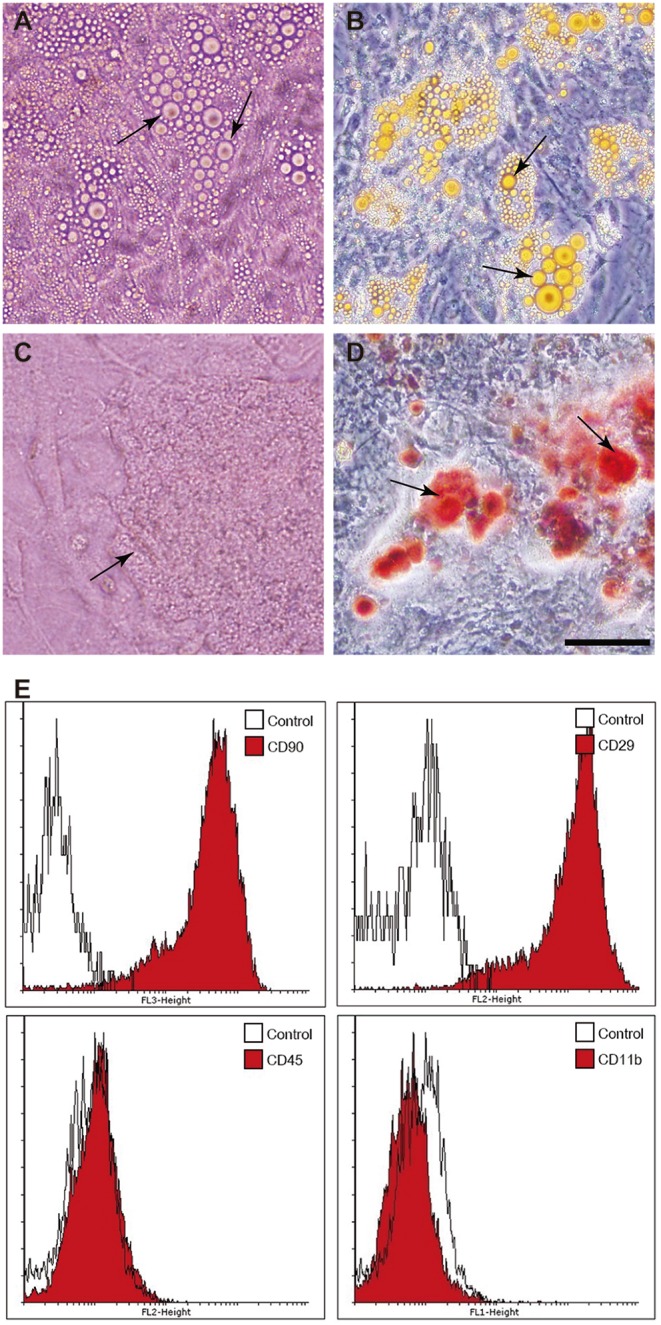
Analysis of the stem cell character of ADSCs extracted from rats. The differentiation of ADSCs is shown in light microscopy at 200X magnification. A. Cells differentiated into adipocytes showing the lipid vacuoles unstained. B. Cells differentiated into adipocytes showing the lipid vacuoles stained with Oil Red. C. Cells differentiated into osteoblasts showing the bone matrix in phase contrast. D. Cells differentiated into osteoblasts showing the bone matrix stained by Alizarin Red. A–D: Scale bar = 100 µm. E. Immunophenotyping analysis of surface marker expression in ADCSs. Flow cytometry histograms show the expression (shaded) of selected molecules (CD90, CD29, CD11b and CD45) by ADSC populations compared with controls (unshaded peaks).

Adherent cells, derived from samples of adipose tissue of rats, showed elongated fusiform morphology with a central nuclei. Cells could be maintained in culture for at least 18 passages. The multipotency of the populations studied was demonstrated by incubating the cells in media promoting differentiation into the osteogenic or adipogenic lineage. ADSCs differentiated into osteoblasts or adipocytes, as evidenced by calcium-rich mineralized matrix deposit or by the presence of intracellular lipid droplets, respectively (Figure 1A–D). The analysis of phenotype of ADSCs used in this study, by flow cytometry, showed that these cells express CD90 (glycosylphosphatidylinositol-anchored glycoprotein) and CD29 (Integrin β1 chain) and were negative for hematopoietic markers, CD11b and CD45 (Figure 1E). According to the literature, the capacity of differentiation and self-replication, as well as adherence on plastic, are parameters acceptable to the confirmation of the identity of MSCs [16], [17], [18].

### CMC Toxicity

**Figure 2:**
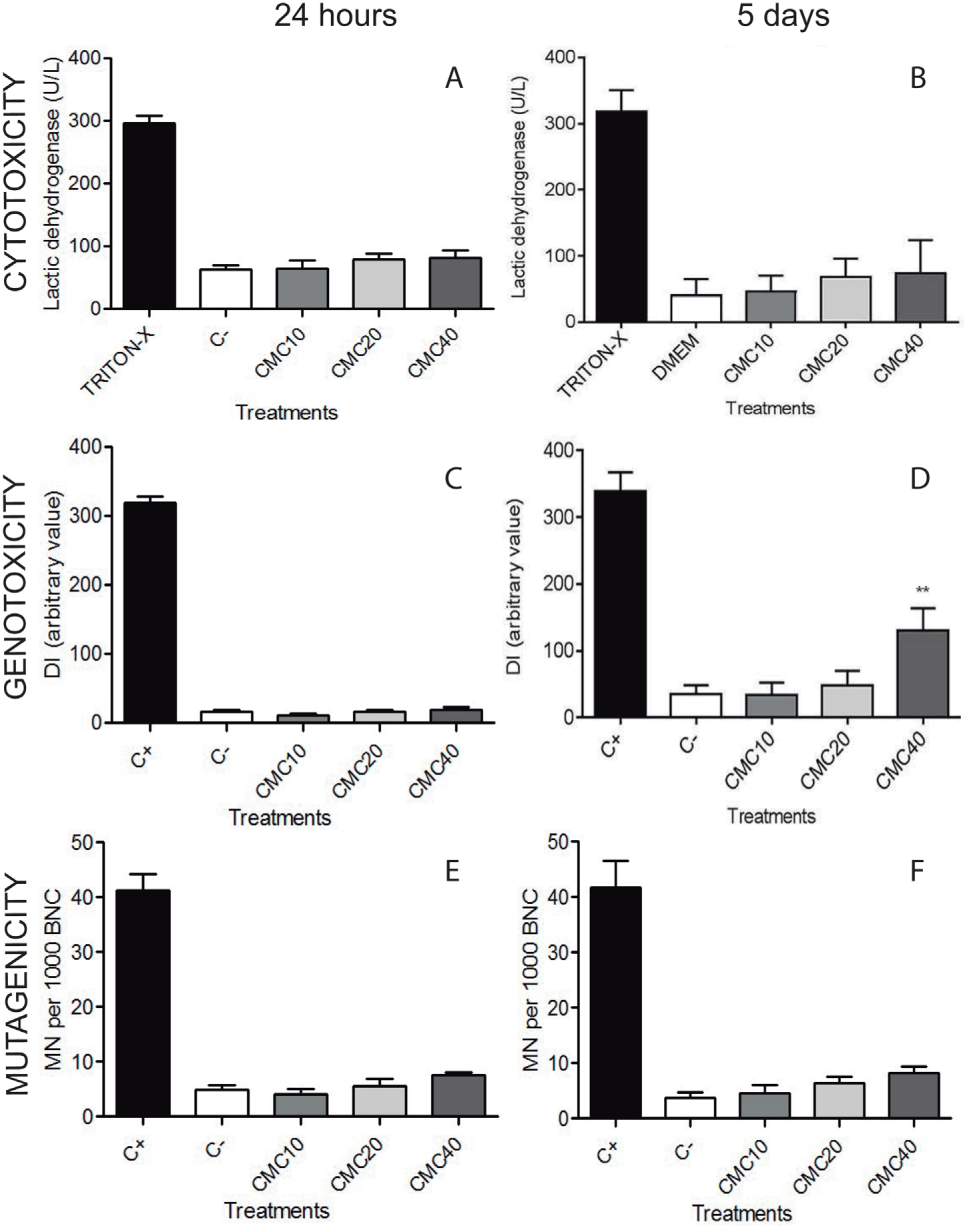
Determination of CMC toxicity on ADSCs cells. The cytotoxicity of CMC (A and B) was evaluated by measuring the LDH enzymatic activity on supernatant of ADSCs. The results are expressed in U/L and were analyzed by one-way ANOVA, followed by Tukey’s multiple comparisons test. The values were considered significantly different from controls, when P<0.05. The potential genotoxicity of CMC (C and D) was analyzed by alkaline comet assay, as described in material and methods. Results are expressed as mean of damage index (DI) ± standard deviation of three independent experiments performed in triplicate. There was statistical significance when comparing CMC in the concentration of 40 mg/mL with the negative control (**P<0.01/One-way ANOVA Tukey’s multiple comparison test). The potential of mutagenicity of CMC (E and F) was evaluated by the *in vitro* cytokinesis-block micronucleus method. The quantification was performed by counting the micronuclei in 1000 binucleated cells (BNCs) with well-preserved cytoplasms. The results were analyzed by one-way ANOVA, followed by Tukey’s multiple comparisons test. The values were considered significantly different from controls, when P<0.05. All toxicity tests were performed in short and long periods of exposition of ADSCs to biomaterial, 24 hours (A, C and E) and 5 days (B, D and F), respectively. CMC: Sodium carboxymethylcellulose; ADSC: Adipose-derived stem cell. MN: micronucleus.

In order to evaluate whether the CMC could damage the cell membrane, we measured the activity of LDH in the supernatant of treated cells for 24 hours or 5 days (Figure 2A and 2B). None of the CMC concentrations tested on ADSCs promoted a significant increase in LDH release. Cells that were cultured for as long as five days in contact with the biomaterial, remained adhered to the plate, similarly to cells grown in normal medium, despite the increased density of the culture medium, as a consequence of the presence of CMC. Cells replicated until reaching 100% of confluence.

The induction of single-strand, double-strand and alkali-labile breaks in ADSCs cells was analyzed using the alkaline comet assay (Figure 2C and 2D). The DNA damage index induced in ADSCs that were grown for 5 days in the presence of CMC was increased at the concentration of 40 mg/mL, when compared to the negative control (DMEM) (Figure 2D). Importantly, this concentration of CMC did not alter the proportions of micronuclei in short (24 hours) or long (5 days) periods (Figures 2E and 2F), suggesting that the small damage observed with the comet assay did not lead to a permanent damage of the cells.

### Healing Parameters

**Figure 3:**
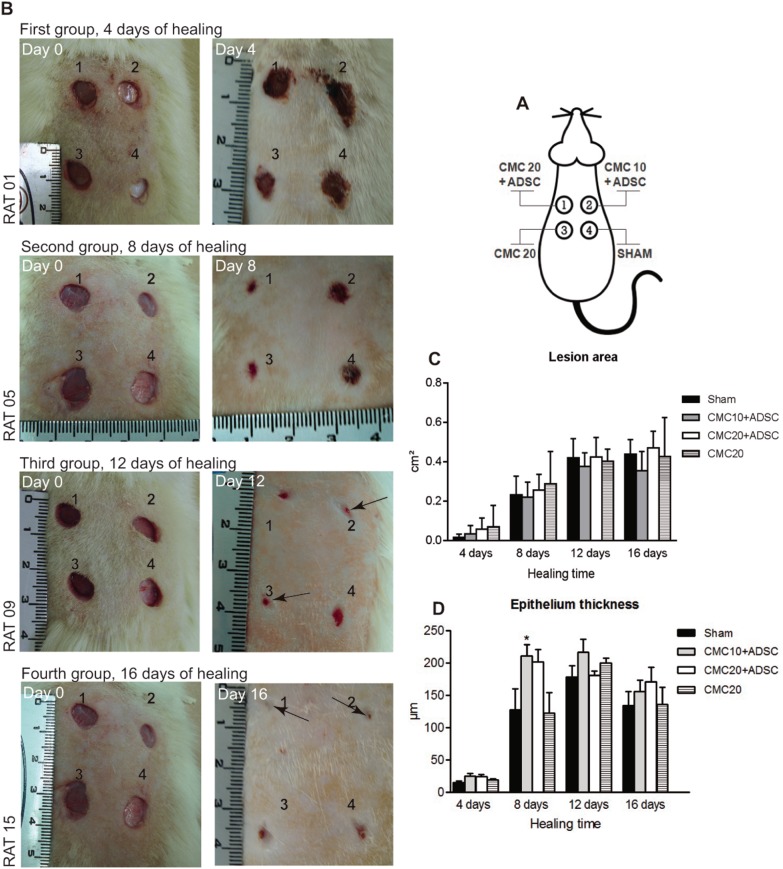
Progression of wound healing. A. Wound model. The scheme shows the four back injuries in a Wistar rat model, comprising the dermis and epidermis layers. The treatments were, in the upper left injury (1): 100 µL of CMC 20 mg/mL+26×10^6^ of ADSCs; upper right (2): 100 µL CMC 10 mg/mL+26×10^6^ of ADSCs; bottom left (3): 100 µL of CMC 20 mg/mL; bottom right (4): Sham. B. The figure is showing the image of one animal, representative of each group, from day 0 to 16, where the closure of wounds was followed and documented daily. Photographs in the left column were taken in day zero, immediately after surgery. In the right column, in the euthanasia day. C. Evaluation of the area of lesions. The graphic shows, in cm^2^, the difference between the size of the initial lesion and the size after different days of healing. There was no difference among the groups. D. Determination of epithelium thickness. Values are expressed in micrometers (µM) and demonstrate a significant increase in the epithelium thickness in the group of animals treated with 10 mg/mL of CMC + ADSCs (lesion 2), with eight days of healing, when compared with control group (injury 4, Sham). (*P<0.05, two-way ANOVA test). CMC: Sodium carboxymethylcellulose; ADSC: Adipose-derived stem cell; Sham: Without treatment.

**Figure 4:**
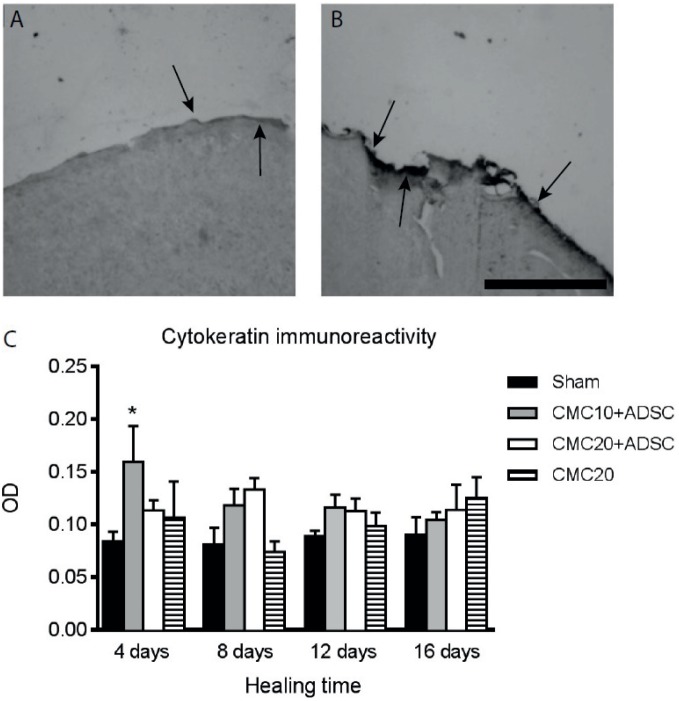
Cytokeratin immunoreactivity in the lesions. A and B. Show representative photomicrographs of the cytokeratin expression in the granulation tissue at the lesion site. The arrows indicate the points where the expression was higher in the lesions: A. Control lesion (Sham); and B. Treated with CMC 10 mg/mL+26×10^6^ ADSCs, with four days of healing. Optical microscopy (400x). C. Quantification of cytokeratin expression. The animal groups were separated by healing time and the different treatments are represented in the legend at right. Analyzes demonstrated statistically significant increase in the immunoreactivity in the first group of animals which had four days of healing, (treated with CMC 10 mg/mL + ADSCs) when compared to Sham. Results are expressed as mean of optical density (OD) (*P<0.05, two-away ANOVA test). CMC: Sodium carboxymethylcellulose; ADSC: Adipose-derived stem cell; Sham: Without treatment. A–B: Scale bar = 10 µm.

The evaluation of the healing process of lesions treated with CMC in the presence or absence of ADSCs was determined by different histological parameters. The observation of lesions during the sixteen days of treatment showed that there was no visible suppurative inflammation. Also, none of the animals became sick or died during the treatment. The representative images of lesions of one animal in each group, at baseline (day zero) and end of healing (day of euthanasia) can be seen in Figure 3B. There was no statistical significance when comparing the sizes of the area of the lesions, treated with CMC, when compared with control lesions (Sham) (Figure 3C). The analysis of histological sections from animals that after treatment had 4 days of healing (first group of animals), showed an epithelium not completely formed to be measured. Among the groups, there was a statistical significant increase, in the second group of animals (8 days in the wound healing treated with CMC 10 mg/mL + ADSCs), when compared with lesions in the control group (Sham) (Figure 3D). The animals euthanized on day 4 (first group) showed a higher expression of cytokeratin with the treatment of CMC 10 mg/mL + ADSCs, when compared with the lesion in negative controls (Sham) (Figures 4 A–C).

**Figure 5:**
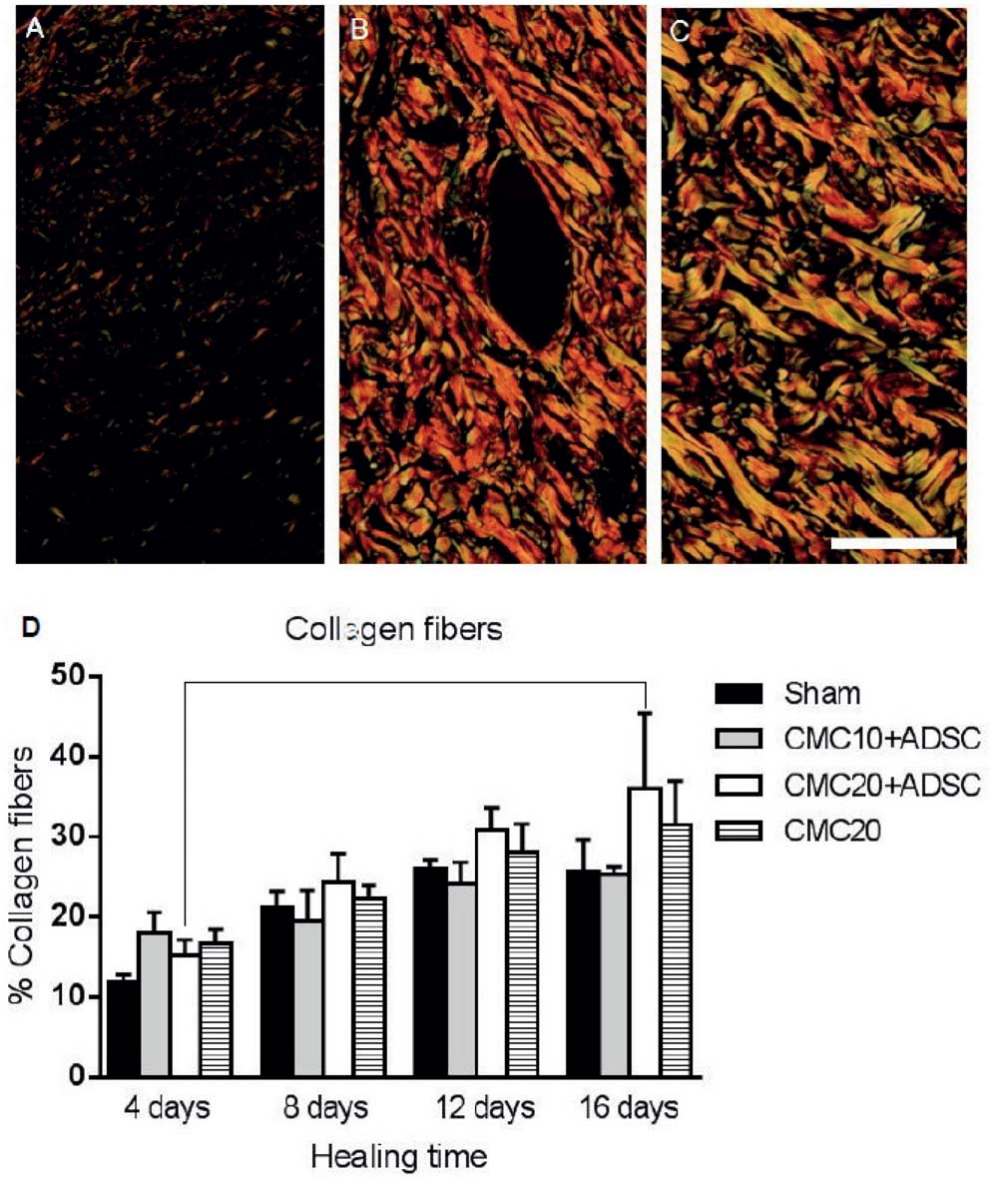
Evaluation of collagen fibers from scar tissues by picrosirius staining. Representative photomicrographs from scar tissues that were subjected to polarization microscopy for visualization of collagen fibers: A. Representative image from the first group with four days of healing. B. From the fourth group with sixteen days of healing, both having received treatment CMC 20 mg/mL+26×10^6^ of ADSCs. C. Shows the collagen fibers in a biopsy of normal skin, not subjected to injury. Optical microscopy. Picrosirius staining (200x). D. The statistical analysis showed significance only in the progression of fibers, when were compared the groups of animals with 4 and 16 days of healing, with the treatment of CMC 20 mg/mL associated with ADSCs. There was no statistical difference in the amount of collagen fibers in any group treated, when compared with control lesions (Sham). CMC: Sodium carboxymethylcellulose; ADSCs: Adipose-derived stem cells; Sham: Without treatment. A–C: Scale bar = 50 µm.

The collagen fibers production increased progressively in the lesions during the sixteen days of treatment. There was a slight increase in collagen with the treatment of CMC 20 mg/mL associated with ADSCs, during the healing process. However the quantification of microphotographs stained by picrosirius technique, showed that there was no statistical difference in the amount of collagen fibers in any treated groups, when compared with Sham (Figure 5D).

## Discussion

CMC is a low cost biomaterial that has been used as dressings for wounds, with the purpose of protection and as an excipient for some drugs [28]. It has been tested as a biomaterial for growing cells [29], [30], however, there is no data evaluating its potential risks and the safe concentrations to be used in therapies. Thus, the first concern in our study was to evaluate the range of safe CMC concentrations to use as a scaffold in cell therapy. For this purpose we performed cytotoxic, genotoxic and mutagenic assays, before testing it in the injured area of an animal. The results showed that there was no significant toxicity on cells cultured with CMC for five days, considering that concentrations of 10 to 40 mg/mL did not cause a significant membrane rupture.

The alkaline comet and micronucleus have been the most commonly used assays for the routine screening of potential genotoxic agents [22]. While the alkaline version of the comet assay [31] detects primary and repairable DNA damage, the micronucleus test detects DNA lesions after their fixation into chromosome mutations. Our results have shown that only at the highest concentration of CMC tested (40 mg/mL), after five days the ADSCs presented increase in DNA damage. However, this damage seems to be repaired, considering that the micronucleus assay did not detect chromosomal damage in ADSCs with the CMC concentrations from 10–40 mg/mL, even with five days of treatment. These results suggest that CMC should be used up to 20 mg/mL.

The epithelization process is an important event for wound healing, ensuring that the new epithelium is formed [32]. In this study, the epithelization rates were improved by CMC containing ADSCs. The cell differentiation and secretion of paracrine factors have been reported to justify the mechanisms by which MSCs contributed to repair damaged tissues [33]. Different molecules are involved in signaling pathways of ADSCs, such as, basic FGF (fibroblast growth factors), VEGF (vascular endothelial growth factor) and PDGF-A (platelet-derived growth factor) [12]. The early development of granulation tissue suggests that the biological events that characterize the dynamic healing are occurring more rapidly, in the group that received treatment of CMC 10 mg/mL associated with ADSCs. An increase in cytokeratin expression, between treated and untreated lesions, suggests a highly active biochemical and bioactive epithelial response, indicative of a migratory epithelium and migratory keratinocyte phenotype, common to the cutaneous wound healing response [34].

MSCs can modulate the host immune responses by changing macrophage, inducing regulatory T cells and inhibiting TH17 formation by both, direct cell-cell contact and indirect mechanisms [35]. This immunomodulatory property could be indirectly related to the epithelization process, since activated macrophages release expressive amounts of FGF [36], a cytokine involved in epithelial proliferation process [37]. A significant increase in cytokeratin expression was observed only in the group of animals, where the healing process occurred by four days. Considering that the therapeutic intervention occurred only once, after the surgery, these results could explain why the animals whose time of healing was 8, 12 and 16 days did not present a significant increase.

The primary route of wound healing in human beings is the formation of granulation tissue and re-epithelialization. Granulation tissue is essential, since it is formed on the surface of the lesion, protecting, nourishing and supporting fibroblasts, new capillaries, and cells from the immune system [38]. The histological slices of lesions stained with HE from animals in the first group (animals with four days of healing), showed a mix of cells, consisting mainly in fibroblasts and leukocytes, which characterize the granulation tissue in its first stage. In the second group, (animals with eight days of healing) the new epithelium observed inside of the wound, was clearly different from the first group, presenting the stratified epithelium. This group also presented an increased epithelium thickness in the lesions that received a concentration of 10 mg/mL of CMC associated with the ADSCs. Interestingly, this result is in accordance with the cytokeratin expression being higher in the group where the healing time was 4 days, with the same treatment. This suggests that during the first 4 days there was an increase in the epithelial cell proliferation, which in the next four days originated a new epithelium layer, capable of being measured and significantly different from the Sham group. Considering that four days of healing were not enough to form a new epithelium, the eight days healing group was the best for this analysis, because the other groups with longer healing time (12 and 16 days), already presented the new epithelium completely formed, independently of treatments.

Although there was a significant increase in the proliferation of epithelial cells in the lesions, with four days of healing, and increased epithelium thickness, in lesions with eight days of healing, when treated with CMC 10 mg/mL plus ADSC, there was no significant difference in the measurements in the injury area when compared to Sham. This improvement of healing at the histological level may be important for large wounds, which would be the ones in which this kind of therapy would be used in the future.

Collagen plays a key role in the wound healing process. The control of its production is a complex process involving a fine balance between its synthesis and degradation [39]. Our analysis by picrosirus stain showed that the treatments did not increase the number of these fibers in comparison with controls (Sham), even in the groups where the epithelium thickness and cytokeratin expression were improved by the treatment. This could be important, because in the healing process, when there is an overproduction of collagen, a fibrosis injury occurs, leading to loss of tissue function and leaving a compromised aesthetic aspect [40], [41]. It is possible that ADSCs regulate the synthesis of collagen in the lesion, by releasing anti-fibrogenic molecules [39]. Taken together, the results suggest that CMC in association with the ADSCs contributed to wound healing in the *in vivo* model studied. Despite promising, this new therapy will need further studies to better investigate the basic mechanisms behind this process, and thus be adapted to clinical applications.

Finally, the results presented here demonstrate an important application for CMC as a biomaterial that presents the properties required for ADSCs growth at concentrations of 20 mg/mL or below. In addition, we showed that CMC at 40 mg/mL has some genotoxic effects, but which do not translate into severe chromosomal damage leading to micronuclei. This can open new avenues for the exploration of the potential pharmaceutical uses of CMC in therapies.
